# Timing and time perception: A selective review and commentary on recent reviews

**DOI:** 10.3389/fpsyg.2014.00648

**Published:** 2014-07-29

**Authors:** Richard A. Block, Simon Grondin

**Affiliations:** ^1^Department of Psychology, Montana State UniversityBozeman, MT, USA; ^2^École de psychologie, Université LavalQuébec City, QC, Canada

**Keywords:** time perception, attention, animals, humans, commentary

A clear example of the progress in the field of timing and time perception could be obtained by contrasting two articles published 30 years apart in the influential *Annual Review of Psychology* (ARP): one by Fraisse (1984), and one by Allman et al. (2014). The fact that there was one author 30 years ago, and a group of authors now, is a tangible sign of the contemporary way of approaching scientific research. In his review, Fraisse emphasized the distinction between time perception and time estimation; in their review, Allman et al. focused on the internal clock and the cerebral bases of timing and time perception.

Fraisse's review was published when a very important event happened in the field of timing and time perception: a conference was held in New York, in 1983, where researchers from both human and animal time perception met to communicate with one another. The conference led to the publication of the classical book edited by the late John Gibbon and the late Lorraine Allan (Gibbon and Allan, 1984). This meeting probably catalyzed the research on timing and time perception, especially the one emphasizing the scalar expectancy theory and, more generally speaking, the internal clock perspective, a clock described as a pacemaker-counter device.

It is somewhat surprising that there was no mention in Fraisse (1984) of this promising (to say the least) pacemaker-counter perspective, which was already available in the human timing literature (Creelman, 1962; Treisman, 1963). Moreover, the modest portions of information in Fraisse dedicated to the cerebral bases of timing exemplify the gap between the contemporary research in the field and the state of the literature 30 years ago.

With its emphasis on neuroscience literature (e.g., brain areas, cortical circuits, pharmacological effects, and pathologies), Allman et al. wrote an important, well-structured, and interesting state-of-the-art review on the cerebral bases of the time perception mechanisms. It is a bit surprising though that the scalar property is taken for granted, given actually Fraisse's fundamental distinction between time perception and time estimation, a distinction that could find some echoes in the limitation of the stability of the Weber fraction for time (see Figure 3 in Gibbon et al., 1997; or, for instance, Grondin, 2001, 2010b, 2012, 2015). Moreover, assuming the linearity between psychological and physical time (psychophysical law) remains disputable (Eisler, 1976).

By emphasizing the internal clock perspective, it was not possible for Allman et al. (2014) to refer to other recent developments in the field. Amongst the portions of the literature the reader might want to consider, there is one on retrospective timing (Block and Zakay, 1997; Tobin et al., 2010). There is also some interesting research (e.g., Boltz, 1998; Brown, 2008) offering a purely cognitive explanation of psychological time and timing—without reference to an internal clock (see reviews by Block et al., 1999, 2010; Block, 2003). Even within the perspective of an internal clock, the attentional-gate model (see for example, Zakay and Block, 1995 and later articles), which in an extension of the scalar expectancy theory, is worth mentioning.

Indeed, with the large increase of research in the field of timing and time perception in the Twenty-first century, it is not surprising to see so many recent special issues of journals on this topic, or close variants of them. The explosion is such that researchers have written a large number of recent review articles (see Table 1). This was partly described in an annotated bibliography on “Time Perception” (Block and Hancock, 2013). Another tangible sign of the vitality of this research field is exemplified by a large COST grant funded by the E.U. (title: “Time In MEntaL activitY,” or “TIMELY”) and the resulting founding of the Brill's new scientific journal dedicated to the psychology of time, Timing and Time Perception, co-edited by Meck et al.

**Table 1:** **Selected list (in reverse chronological order) of reviews since 2010 on the psychology of time**.

**Type**	**Authors**	**Year**	**Title**
Book	Merchant and de Lafuente	2015	Neurobiology of interval timing
SI	Medina et al.	2014	Advances in modern mental chronometry
Book	Vatakis and Allman	2014	Time distortions in mind: temporal processing in clinical populations.
Rev	Allman et al.	2014	Properties of the internal clock: first- and second-order principles of subjective time
Rev	Block and Gruber	2014	Time perception, attention, and memory: a selective review
SI	Broadway et al.	2014	The long and short of mental time travel– self-projection over time-scales large and small
SI	Buhusi	2014	Associative and temporal learning: New directions
Book	Lloyd and Arstila	2014	Subjective time: the philosophy, psychology, and neuroscience of temporality
Rev	Matthews and Meck	2014	Temporal perception: the bad news and the good
SI	Tucci et al.	2014	Timing in neurobiological processes: from genes to behavior compiled
SI	Vatakis and Ulrich	2014	Temporal processing within and across senses (two *Acta Psychologica* special issues)
Bib	Block and Hancock	2013	Time perception (annotated bibliography)
SI	Coull et al.	2013	How does the brain process time?
Rev	Merchant et al.	2013	Neural basis of the perception and estimation of time
Rev	Wittmann	2013	The inner sense of time: how the brain creates a representation of duration
Rev	Allman and Meck	2012	Pathophysiological distortions in time perception and timed performance
Rev	Hancock and Block	2012	The psychology of Time: a view backward and forward
SI	Meck et al.	2012	Interval timing and time-based decision making
Rev	Coull et al.	2011	Neuroanatomical and neurochemical substrates of timing
Rev	Gorea	2011	Ticks per thought or thoughts per tick? A selective review of time perception with hints on future research
SI	Vatakis et al.	2011	Multidisciplinary aspects of time and time perception
Rev	Block et al.	2010	How cognitive load affects duration judgments: a meta-analytic review
Rev	Grondin	2010a	Timing and time perception: a review of recent behavioral and neuroscience findings and theoretical directions

Book is an edited book. Rev is a review article. SI is a special issue. Bib is a bibliography.

In conclusion, being a researcher in the field of timing and time perception has never been as exciting as it is at present, given the growth of its popularity, which has been enhanced by the arrival of contributions from neuroscientists. This excitement could be extended if one considers psychological time in an even larger perspective, or larger scale from the memory for the past events (Friedman, 1993) to the capacity to predict the duration of future events (Roy et al., 2005).

## Conflict of interest statement

The authors declare that the research was conducted in the absence of any commercial or financial relationships that could be construed as a potential conflict of interest.

## References

[B40] Allman M. J. Meck W. H. (2012). Pathophysiological distortions in time perception and timed performance. Brain 135, 656–677 10.1093/brain/awr210 21921020 PMC3491636

[B1] Allman M. J. Teki S. Griffiths T. D. Meck W. H. (2014). Properties of the internal clock: first- and second-order principles of subjective time. Annu. Rev. Psychol. 65, 743–771 10.1146/annurev-psych-010213-115117 24050187

[B2] Block R. A. (2003). Psychological timing without a timer: the roles of attention and memory, in Time and Mind II, ed Helfrich H. (Cambridge, MA: Hogrefe & Huber), 41–60

[B3] Block R. A. Gruber R. P. (2014). Time perception, attention, and memory: a selective review. Acta Psychol. 149, 129–133 10.1016/j.actpsy.2013.11.003 24365036

[B4] Block R. A. Hancock P. A. (2013). Time perception, in Annotated Bibliography (Oxford Online Bibliographies), 284–295 http://www.oxfordbibliographies.com

[B5] Block R. A. Hancock P. A. Zakay D. (2010). How cognitive load affects duration judgments: a meta-analytic review. Acta Psychol. 134, 330–343 10.1016/j.actpsy.2010.03.006 20403583

[B6] Block R. A. Zakay D. (1997). Prospective and retrospective duration judgments: a meta-analytic review. Psychon. Bull. Rev. 4, 184–197 10.3758/BF03209393 21331825

[B7] Block R. A. Zakay D. Hancock P. A. (1999). Developmental changes in human duration judgments: a meta-analytic review. Dev. Rev. 19, 183–211 10.1006/drev.1998.0475

[B8] Boltz M. G. (1998). The processing of temporal and nontemporal information in the remembering of event durations and musical structure. J. Exp. Psychol. Hum. Percept. Perform. 24, 1087–1104 10.1037/0096-1523.24.4.1087 9706710

[B39] Broadway J. M. Zedelius C. Schooler J. Grondin S. (2014). The long and short of mental time travel- self-projection over time-scales large and small. Front. Psychol. Perception Science. 10.3389/fpsyg.2015.00668 PMC4438603 26042080

[B9] Brown S. W. (2008). Time and attention: review of the literature, in Psychology of Time, ed Grondin S. (Bingley: Emerald), 111–138

[B10] Buhusi C. V. (2014). Associative and temporal learning: new directions. Behav. Process. 101, 1–3 10.1016/j.beproc.2014.01.005 24560413

[B11] Coull J. T. Cheng R.-K. Meck W. H. (2011). Neuroanatomical and neurochemical substrates of timing. Neuropsychopharmacology 36, 3–25 10.1038/npp.2010.113 20668434 PMC3055517

[B12] Coull J. T. Van Wassenhove V. Coslett H. B. (eds.). (2013). How does the brain process time? Neuropsychologia 51, 187–38423022107

[B13] Creelman C. D. (1962). Human discrimination of auditory duration. J. Acoust. Soc. Am. 34, 582–593 10.1121/1.1918172

[B14] Eisler H. (1976). Experiments on subjective duration 1878–1975: a collection of power function exponents. Psychol. Bull. 83, 185–200 10.1037/0033-2909.83.6.1154 996212

[B15] Fraisse P. (1984). Perception and estimation of time. Annu. Rev. Psychol. 35, 1–36 10.1146/annurev.ps.35.020184.000245 6367623

[B16] Friedman W. J. (1993). Memory for the time of past events. Psychol. Bull. 113, 44–66 10.1037/0033-2909.113.1.44

[B17] Gibbon J. Allan L. G. (eds.). (1984). Annals of the New York Academy of Sciences. *Vol. 423. Timing and Time Perception* New York, NY: New York Academy of Sciences

[B18] Gibbon J. Malapani C. Dale C. L. Gallistel C. (1997). Toward a neurobiology of temporal cognition: advances and challenges. Curr. Opin. Neurobiol. 7, 170–184 10.1016/S0959-4388(97)80005-0 9142762

[B42] Gorea A. (2011). Ticks per thought or thoughts per tick? A selective review of time perception with hints on future research. J. Physiol. 105, 153–163 10.1016/j.jphysparis.2011.09.008 21963529

[B19] Grondin S. (2001). From physical time to the first and second moments of psychological time. Psychol. Bull. 127, 22–44 10.1037/0033-2909.127.1.22 11271754

[B20] Grondin S. (2010a). Timing and time perception: a review of recent behavioral and neuroscience findings and theoretical directions. Atten. Percept. Psychophys. 72, 561–582 10.3758/APP.72.3.561 20348562

[B21] Grondin S. (2010b). Unequal Weber fraction for the categorization of brief temporal intervals. Atten. Percept. Psychophys. 72, 1422–1430 10.3758/APP.72.5.1422 20601721

[B22] Grondin S. (2012). Violation of the scalar property for time perception between 1 and 2 seconds: evidence from interval discrimination, reproduction, and categorization. J. Exp. Psychol. Hum. Percept. Perform. 38, 880–890 10.1037/a0027188 22390291

[B23] Grondin S. (2015). About the (non)scalar property for time perception, in Neurobiology of Interval Timing, eds Merchant H. de Lafuente V. (New York, NY: Springer).

[B41] Hancock P. A. Block R. A. (2012). The psychology of time: A view backward and forward. Am. J. Psychol. 125, 267–274 10.5406/amerjpsyc.125.3.0267 22953687

[B24] Lloyd D. Arstila V. (eds.). (2014). Subjective Time: The Philosophy, Psychology, and Neuroscience of Temporality. Cambridge, MA: MIT Press

[B25] Matthews W. J. Meck W. H. (2014). Time perception: the bad news and the good. WIREs Cogn. Sci. 5, 429–446 10.1002/wcs.1298 PMC4142010 25210578

[B26] Meck W. H. Doyère V. Gruart A. (2012). Interval timing and time-based decision making. Front. Integr. Neurosci. 6:13 10.3389/fnint.2012.00013 22479240 PMC3315844

[B27] Medina J. M. Wong W. Díaz J. A. Colonius H. (2014). Advances in modern mental chronometry. Front. Hum. Neurosci. 10.3389/fnhum.2015.00256 PMC4422014 25999843

[B28] Merchant H. de Lafuente V. (eds.). (2015). Neurobiology of Interval Timing. New York, NY: Springer

[B29] Merchant H. Harrington D. L. Meck W. H. (2013). Neural basis of the perception and estimation of time. Annu. Rev. Neurosci. 36, 313–336 10.1146/annurev-neuro-062012-170349 23725000

[B30] Roy M. M. Christenfeld N. J. S. McKenzie C. R. M. (2005). Underestimation of future duration: memory incorrectly used or memory bias. Psychol. Bull. 131, 738–756 10.1037/0033-2909.131.5.738 16187856

[B31] Tobin S. Bisson N. Grondin S. (2010). An ecological approach to prospective and retrospective timing of long durations: a study involving gamers. PLoS ONE 5:e9271 10.1371/journal.pone.0009271 20174648 PMC2822850

[B32] Treisman M. (1963). Temporal discrimination and the indifference interval: implications for a model of the “internal clock.” Psychol. Monogr. 77, 576 10.1037/h0093864 5877542

[B33] Tucci V. Buhusi C. V. Gallistel R. Meck W. H. (2014). Timing in neurobiological processes: from genes to behaviour compiled. Philos. Trans. R. Soc. B 369:20120470 10.1098/rstb.2012.0470 PMC3895994 24446503

[B34] Vatakis A. Allman M. J. (eds.) (2014). Time Distortions in Mind: Temporal Processing in Clinical Populations. Boston, MA: Brill Academic Publishers

[B35] Vatakis A. Esposito A. Giagkou M. Cummins F. Papadelis G. (2011). Multidisciplinary Aspects of Time and Time Perception. Vol. 6789. Lecture Notes in Computer Science, Berlin: Springer 10.1007/978-3-642-21478-3

[B36] Vatakis A. Ulrich R. (eds.). (2014). Temporal processing within and across senses. Acta Psychol. (Amst.) 147(pt 1 and 2), 149. 10.1016/j.actpsy.2014.01.001 24507734

[B37] Wittmann M. (2013). The inner sense of time: how the brain creates a representation of duration. Nat. Rev. Neurosci. 14, 217–223 10.1038/nrn3452 23403747

[B38] Zakay D. Block R. A. (1995). An attentional-gate model of prospective time estimation, in Time and the Dynamic Control of Behavior, eds Richelle M. Keyser V. D. d'Ydewalle G. Vandierendonck A. (Liège, Belgium: Université de Liège), 167–178

